# A Sound and Vibration Fusion Method for Fault Diagnosis of Rolling Bearings under Speed-Varying Conditions

**DOI:** 10.3390/s23063130

**Published:** 2023-03-15

**Authors:** Haibo Wan, Xiwen Gu, Shixi Yang, Yanni Fu

**Affiliations:** 1 School of Mechanical and Automobile Engineering, Zhejiang University of Water Resources and Electric Power, Hangzhou 310018, China; wanhb@zjweu.edu.cn; 2 The State Key Laboratory of Fluid Power and Mechatronic Systems, School of Mechanical Engineering, Zhejiang University, Hangzhou 310027, China; guxiwen@zju.edu.cn; 3 The Key Laboratory of Advanced Manufacturing Technology of Zhejiang Province, School of Mechanical Engineering, Zhejiang University, Hangzhou 310027, China; 4 Hangzhou Steam Turbine Co., Ltd., Hangzhou 310022, China; fuyn@htc.cn

**Keywords:** fault diagnosis, convolutional neural network, sound and vibration fusion, multiple scales, rolling bearing

## Abstract

The fault diagnosis of rolling bearings is critical for the reliability assurance of mechanical systems. The operating speeds of the rolling bearings in industrial applications are usually time-varying, and the monitoring data available are difficult to cover all the speeds. Though deep learning techniques have been well developed, the generalization capacity under different working speeds is still challenging. In this paper, a sound and vibration fusion method, named the fusion multiscale convolutional neural network (F-MSCNN), was developed with strong adaptation performance under speed-varying conditions. The F-MSCNN works directly on raw sound and vibration signals. A fusion layer and a multiscale convolutional layer were added at the beginning of the model. With comprehensive information, such as the input, multiscale features are learned for subsequent classification. An experiment on the rolling bearing test bed was carried out, and six datasets under various working speeds were constructed. The results show that the proposed F-MSCNN can achieve high accuracy with stable performance when the speeds of the testing set are the same as or different from the training set. A comparison with other methods on the same datasets also proves the superiority of F-MSCNN in speed generalization. The diagnosis accuracy improves by sound and vibration fusion and multiscale feature learning.

## 1. Introduction

The rolling bearing is a critical component of rotating machinery such as the wind turbine, the hydraulic turbine, and the aero-engine. Rolling bearing faults are the leading cause of the failure of rotating machinery, bringing economic losses and even unplanned downtime. The fault diagnosis of rolling bearings before catastrophic failure is one of the main concerns of these industries [1] and is essential for the availability and reliability of mechanical systems [2]. 

The traditional fault diagnosis of rolling bearings is constructed by extracting fault characteristic frequencies (FCFs) from monitoring signals [2,3]. FCFs can be calculated by the geometric parameters and running speeds of the rolling bearings. However, most of the rolling bearings work under speed-varying conditions in practice, including the run-up and shutdown of machines and speed fluctuation due to variable loads. When the speed varies, the FCF changes with the speed, and the spectrum of the nonstationary signal shows a smearing phenomenon. Order analysis is a commonly employed approach that deals with time-varying FCFs [4]. The effect of speed variation can be removed by resampling the raw signal in the angular domain, and the FCFs are converted to the fault characteristic orders (FCOs), which are constant. Order analysis relies heavily on rotational frequency, which can be obtained by installing an auxiliary tachometer [5] or estimated from the existing signals [6,7]. In addition, many studies have focused on extracting time-varying fault characteristics directly through time-frequency analysis. Several methods have been proposed to obtain high-quality time-frequency representations with fine resolution and better energy concentration [8,9,10].

Although these methods have proved effective under speed-varying conditions, prior knowledge is necessary to calculate the FCFs or FCOs. Deep learning (DL) algorithms have recently gained increasing interest and have proved their ability to diagnose faults more automatically and intelligently [11,12]. As a widely studied DL method, the convolutional neural network (CNN) has obtained promising results in fault diagnosis [13], especially for the rolling bearings [14]. With sparse connectivity and shared weights, CNN can better learn the deep features while, to some extent, avoiding overfitting and consuming fewer computational resources. Some of the literature concerns the use of CNN as an automatic feature extractor followed by other classifiers [15]. However, most attention has focused on end-to-end fault diagnosis combining the feature learning and classification process [16]. 

Studies on the fault diagnosis of rolling bearings under variable speed conditions using CNN represent a growing field. Huang et al. [17] constructed the datasets including six different operation conditions to study the performance of CNN when treating nonstationary signals. Similarly, multiple-speed working conditions were considered in Refs. [18,19,20]. CNN-based model validation experiments were also performed using datasets under speed-up conditions [21,22]. Usually, the monitoring data points are divided into two datasets using a sliding window or by random sampling. One of the datasets was used for training the fault diagnosis model, while the other was the testing dataset that verified the model performance. Though variable speed is investigated in these studies, the testing samples are in the same operating conditions as the training samples. However, this is not the case in the real world, where the operating speed of the rolling bearings is more changeable. It is generally challenging to gather comprehensive data covering all the speeds for training, especially during the early operation of the equipment. Therefore, more attention should be paid to the generalization of the model. This paper carefully evaluates the adaptation ability to cross different speed domains for the fault diagnosis problem of roller bearings under speed-varying conditions.

Some examples in the literature attempt to deal with the cross-domain problem by transfer learning algorithms, but the transfer of the model is relatively complicated, and data from the target domain are essential for training a model [23,24]. A CNN model could realize the cross-domain transfer of working speeds without domain adaptation treatments. Wang et al. [25] proved the robustness of the CNN model to missing data by removing some data points in the training datasets, and the maximum missing data rate in their work was 40%. Wang et al. [26] tested the CNN-based model with the training and testing datasets from different working speeds. However, multiple constant speeds instead of time-varying speeds were investigated, and the time series of the raw signals were converted into feature images before they were placed into the CNN model. In this paper, we are trying to address the problem of the fault diagnosis of rolling bearings under speed-varying conditions using an improved CNN model that works directly on the raw signals. The speed-varying conditions include different situations. On the one hand, the samples could be at multiple constant speeds or speeds that change with time; on the other hand, the training and testing samples could be at the same or different speeds. A novel CNN architecture, namely the fusion multiscale convolutional neural network (F-MSCNN), was proposed to address speed-varying problems. Compared with the original CNN model, a fusion layer and a multiscale layer were added to the model to enhance the model’s generalization ability.

One of the outstanding advantages of CNN is its capability to process multidimensional data so that the input of a CNN model can contain heterogeneous information [20]. CNN with data fusion has proved beneficial in improving the fault diagnosis accuracy of rolling bearings. Comprehensive information on the faults can be gathered by fusing the monitoring signals. Related work includes fusing the vibration data of multi-directions [27], fusing the vibration signals mounted on different locations [28], and the fusion of vibration signals and the current signal [29]. The sound signal analysis provides a contactless and effective solution for the fault diagnosis of rotating machines [30,31]. Incipient fault detection and classification can be achieved using sound data collected in a noisy environment [32]. Inspired by these works, the fusion of the sound and vibration data of rolling bearings is studied in this paper to obtain complementary information on the faults. Instead of simply stitching the multiple signals, a two-dimension fusion layer was added at the beginning of the network to obtain the common and characteristic information of the sound and vibration signal.

In addition, convolutional kernels of different sizes were adopted in the proposed model. The concept of multiscale convolution comes from the CNN model codenamed Inception [33]. Convincing results have been obtained with several Inception modules introduced in the original CNN model [34]. Multiscale learning was also performed by parallel convolutional pathways and feature-level fusion at the later layers [35,36]. Such structures could achieve good performance but increase the parameters of the model. Huang et al. [17] used a multiscale convolution at the beginning of the model to find the sensitive bands in the frequency domain of different resolutions. The fused sound and vibration signals also contained heterogeneous information, including multiple time characteristics at varying speeds. Therefore, this paper added a multiscale layer after the fusion layer to learn the heterogeneous features. The features were then concatenated and put into other feature learning layers to realize the end-to-end automatic fault diagnosis.

This paper primarily includes the following contributions:

(1) The data fusion, feature extraction, and fault classification processes are combined into an end-to-end automated procedure. The proposed model works directly on the raw signals of sound and vibration.

(2) The proposed method has a strong generalization capacity. In model validation, different speed-varying situations are taken into consideration. Datasets are constructed by samples at multiple constant speeds or speeds changing with time. The speeds of the testing samples are the same as or are unknown to the training sample. The proposed method can achieve high and stable accuracy under various datasets, especially fault diagnosis when crossing different speed domains.

(3) The F-MSCNN method adds a fusion layer and a multiscale convolutional layer to enhance the model performance under speed-varying conditions. The adaptive fusion of the sound and vibration data is realized at the beginning of the network to input comprehensive information to the model. The multiscale convolution operators could increase the depth and width of the network to learn more comprehensive features.

(4) The feature maps learned by F-MSCNN are visualized to reveal the inner feature learning mechanism.

The layout of this paper consists of five sections. Section 2 formulates the traditional CNN method. Section 3 describes the proposed F-MSCNN in detail. A verification experiment is represented in Section 4 with a discussion of the diagnosis results, including visualization analysis, computational costs analysis, and comparison with other CNN-based methods and machine learning methods. Finally, the conclusions and further work are shown in Section 5.

## 2. Convolutional Neural Networks

A convolutional neural network is a specialized kind of neural network that uses the convolution operation in at least one layer in place of a general matrix multiplication [37]. A typical structure of CNN includes an input layer, convolution layer, pooling layer, and fully connected layer. The convolutional layer adopts filter kernels to convolve the input local regions and generate the local features. The desired features can be reorganized and extracted from the raw input data through a series of convolutions. The *j*th output feature matrix of the lth convolutional layer $X_{j}^{l}$ is described as:

(1) $$X_{j}^{l}=\Phi \left(Z_{j}^{l}\right)=\Phi \left(\sum_{i}X_{i}^{l-1}*W_{j}^{l}+b_{j}^{l}\right)$$

where * is the convolution operator and $X_{i}^{l-1}$ is the *i*th input feature matrix of the layer *l* − 1. $W_{j}^{l}$ and $b_{j}^{l}$ are the weights of the convolution kernel and the biases, respectively. The feature matrix $Z_{i}^{l}$ is the output of the convolution operation. An activation function **Φ** is added to enhance the nonlinear expression ability. A rectified linear unit (ReLU) and sigmoid are commonly used as activation functions.

(2) $$\Phi_{\mathrm{ReLu}}(z)=\{\begin{array}{c} z,z\geq 0\\ 0,z<0 \end{array}$$

(3) $$\Phi_{\mathrm{sigmoid}}(z)=1/\left(1+\exp(-z)\right)$$

The pooling layer is used to reduce the spatial size of the feature map. The pooling operation helps to reduce computational complexity and avoid over-fitting. The pooling layer does not learn features. It performs downsampling by dividing each of the input feature maps into rectangular pooling regions. Max-pooling is the most widely used pooling type. For the one-dimensional max-pooling, given the size of the pooling region *w*, the *j*th max-pooling output of the layer *l* + 1 is presented as:

(4) $$p_{j}^{l+1}(k)=\underset{(k-1)w+1\leq t\leq kw}{\max}\left\{q_{j}^{l}(t)\right\}$$

where $q_{j}^{l}(t)$ is the *j*th output feature map of layer *l*. *t* denotes the *t*th pooling region and t∈[(k−1)w+1,kw]. The maximum of each region is returned.

After several rounds of the convolutional and pooling layer, the fully connected layer is added to generate the high-level feature vector. The fully connected layer multiplies the inputs by a weight matrix and then adds a bias vector. Each neuron of the previous layer is connected to this layer.

(5) $$y_{j}^{l+1}=\Phi \left(\sum_{i=1}W_{j}^{l}X_{i}^{l}+b_{j}^{l}\right)$$

where $W_{j}^{l}$ and $b_{j}^{l}$ indicate the weight and bias, respectively. $X_{i}^{l}$ is the ith input feature map of layer *l*, and the corresponding output is $y_{j}^{l+1}$. For a classification problem, a softmax activation function is applied in the last fully connected layer to recognize the patterns of the feature. The softmax function calculates the probability of each sample under all the possible target classes.

(6) $$p(y_{j}^{l+1})=e^{y_{j}^{l+1}}/\sum e^{y_{j}^{l+1}}$$

## 3. Proposed F-MSCNN

**Figure 1:**
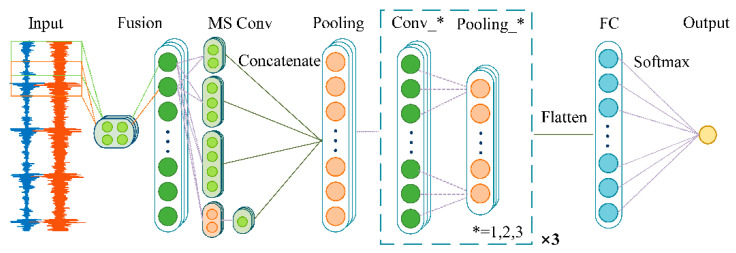
The structure of the proposed F-MSCNN.

This paper proposes a novel CNN-based deep learning approach that is aimed at the fault diagnosis of rolling bearings under speed-varying conditions. The proposed F-MSCNN provides an end-to-end automated procedure to perform more accurate fault classification when the operating speed changes with time. A fusion layer and a multiscale convolution layer were added to better fuse and extract the characteristic information from the raw vibration and sound signals. The structure of F-MSCNN is shown in Figure 1, including an input layer (Input), a fusion layer (Fusion), a multiscale convolution layer (MS Conv), simple feature learning layers with three simple convolutional layers (Conv_1, Conv_2, and Conv_3, respectively), several pooling layers (Pooling), and a fully connected layer (FC).

### 3.1. Fusion of Sound and Vibration

Signal fusion has been proven as an effective way to enhance diagnosis accuracy. A fusion layer was constructed at the beginning of the F-MSCNN model to enrich the condition information and obtain a comprehensive description of the diagnostic object. The sound and vibration signals are two commonly used signals for condition monitoring and the fault diagnosis of rolling bearings. Usually, an accelerometer for collecting vibration signals is installed near the faulty rolling bearing so the abnormality can be captured in a timely manner. The mounting position should be carefully arranged for the accelerometer, and it is better installed on the vibration propagation path not far away from the monitored object to avoid interferences from other vibration sources. The sound signal is homologous to the vibration signal. A microphone was used for sound acquisition, which generally had a wider effective bandwidth to capture more information [38]. Additionally, the installation of the microphone was more convenient and adjustable with non-contact measurements. However, the sound signal could be easily disturbed by ambient noise. In this paper, sound and vibration signals were fused to make the best of both worlds. The sound and vibration signals were put together into a matrix of *m* × 2 as the input of the model, where m is the length of one sample. The fusion process was realized by a two-dimension convolution operation with *k* convolutional kernels of size *n* × 2. Fusion by convolution is also a feature extraction process that can fully obtain the common and distinctive information of sound and vibration signals.

### 3.2. Multiscale Convolution

**Figure 2:**
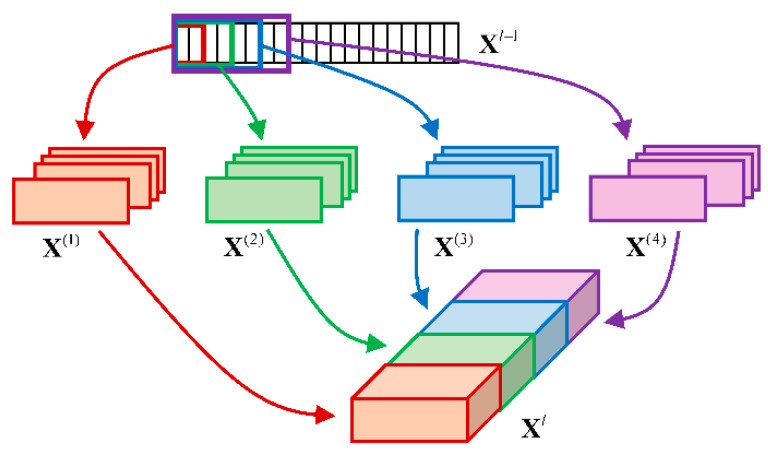
The multiscale layer including convolutions of different scales.

The conventional CNN structure used in fault diagnosis adopts only one kernel in the convolution layer. However, under speed-varying conditions, the inherent characteristics of the signals are diverse at multiple speed scales. A multiscale layer with convolutional kernels of different scales was added in an F-MSCNN model to better extract the features and enhance the diversity and performance of the model. The multiscale layer is illustrated in Figure 2. Four filters of different scales were designed to convolve the fused signal. The results of different filters were padded with zero evenly to the boundary of the input so that the length of the outputs was consistent and different outputs could be concatenated before they were put into the next layer. The final output representation of the multiscale convolution layer is represented as:

(7) $$X^{l}=[X^{\left(1\right)},X^{\left(2\right)},X^{\left(3\right)},X^{\left(4\right)}]$$

where $X^{\left(\cdot \right)}$ is the corresponding representation convolved by the multiscale kernels.

### 3.3. Training of F-MSCNN

The training process of the proposed F-MSCNN is detailed in this section. The cross-entropy between the predicted soft-max output probability distribution and the target label probability distribution is defined as the lost function of the model:

(8) $$L=-\sum_{x}p(x)\log q(x)$$

where *p*(**x**) is the target distribution while *q*(**x**) denotes the estimated distribution of the soft-max layer.

The momentum stochastic gradient descent algorithm was applied to minimize the cost function during the training process to optimize the parameters. The use of momentum can reduce the oscillations of the stochastic gradient descent. The momentum update rule for the weight and bias parameters is:

(9) $$\{\begin{array}{c} V_{dW^{l}}=\beta \cdot V_{dW^{l}}+(1-\beta )dW^{l}\\ W^{l}=W^{l}-\alpha \cdot V_{dW^{l}} \end{array},\{\begin{array}{c} V_{db_{l}}=\beta \cdot V_{db_{l}}+(1-\beta )db_{l}\\ b_{l}=b_{l}-\alpha \cdot V_{db_{l}} \end{array}$$

where *β* is the momentum and *α* is the learning rate. $V_{dW^{l}}$ represents the velocity of the gradient $dW^{l}$. 

Batch normalization (BN) was used to accelerate the training process and reduce the internal covariate shift of the model [39]. $X_{j}$ and $H_{j}$ represent the *j*th input and output of BN, and the operation of BN is denoted by:

(10) $${\tilde{X}}_{j}=\left(X_{j}-E[X_{j}]\right)/\sqrt{\mathrm{Var}[X_{j}]},\;H_{j}=\gamma_{j}{\tilde{X}}_{j}+\beta_{j}$$

where E[⋅] is the expectation and Var [·] is the variance. $\gamma_{j}$ and $\beta_{j}$ represent the scale and shift parameters, respectively.

### 3.4. Fault Diagnosis Using F-MSCNN

**Figure 3:**
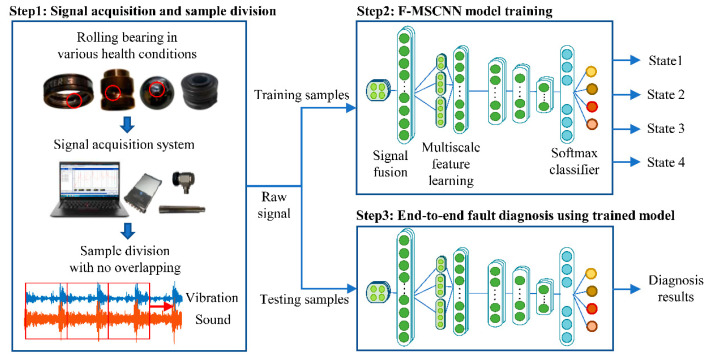
The process of end-to-end fault diagnosis by F-MSCNN.

The process of the fault diagnosis of rolling bearings under speed-varying conditions using F-MSCNN is illustrated in Figure 3. There are mainly three steps, which are summarized as follows.

Step1: Sound and vibration signals from varying speeds of the rolling bearings were collected synchronously. Both of the signals were sliced without overlap into small segments for the following model training, validation, and testing. The two segments of the sound and vibration signals at the same period of time were combined together as an input map. The input was directly put into the model without expert experience or signal processing technologies. 

Step 2: The data map, including sound and vibration data, was used as the input of the F-MSCNN model to establish an end-to-end automated fault diagnosis framework. The output of the model was fault labeled. Information fusion, multi-scale feature extraction, and classification were realized by training the model. The training samples were mixed with multiple speeds but did not need to cover all the operating speeds.

Step 3: The trained model was put into use for fault diagnosis under speed-varying conditions. The testing samples could be in the same working condition as the training samples, which is the common treatment. The testing samples could be in other speed conditions different from the training samples due to the speed generalization ability of the model.

## 4. Method Validation by Experimental Data

### 4.1. Experiment and Dataset Description

**Figure 4:**
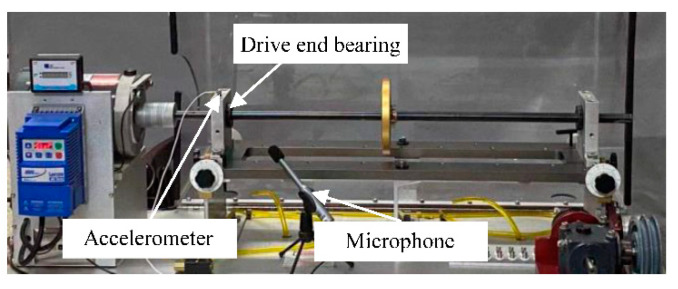
Test bed with faults injected to the driven end bearing.

The proposed approach was verified by an experiment with rolling bearings on the machinery fault simulator, as shown in Figure 4. The same type of deep groove rolling bearings (MB ER 12K) were installed on the drive end and non-driven end. For the drive end bearing, four different conditions were considered in the experiment, including three modes of faults and the normal condition(N). Ball fault (BF), outer race fault (OF), and inner race fault (IF) were injected into the bearing separately. An accelerometer was mounted on the top of the faulty bearing house to gather the vibration signals, while a microphone was placed 20 cm near the faulty bearing to gather the sound signals synchronously. The sampling frequency was 48 kHz.

**Figure 5:**
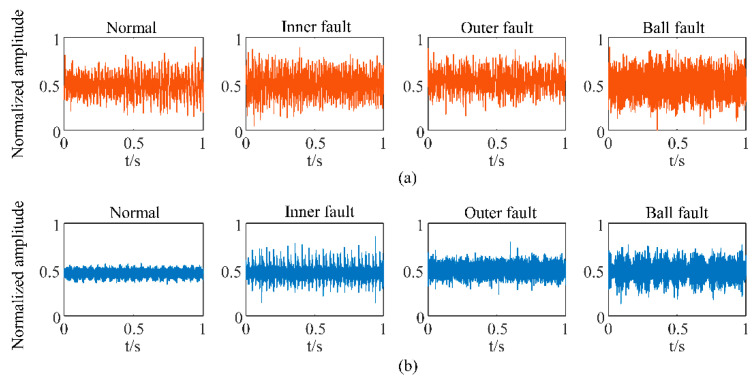
Waveforms of (**a**) sound signals and (**b**) vibration signals for different health conditions of rolling bearing.

For each condition of the bearing, the data were collected at different rotating speeds from 600 to 2400 rpm with an equal interval of 300 rpm, so there were seven working conditions with different speeds. For each constant speed, the sampling time was 30 s. Additionally, another three working conditions with speed decreasing from 2400 to 600 rpm at the rate of 1 Hz/s and 0.5 Hz/s were considered. The sampling time was 30 s and 60 s, respectively. The same health condition under different speeds was treated as one class, and there were four classes in all for classification. Figure 5 shows the time-domain waveforms of the sound and vibration signals for different health conditions under 1200 rpm. It shows that the sound and vibration signals had different behaviors for different health conditions. The amplitude of the sound signal was greater than that of the vibration signal under the same condition. The sound signal seemed to be more susceptible to environmental interference. It is clearly shown in Figure 5b that there are more impact components in the fault signals than under normal conditions. However, it is challenging to distinguish different faults directly from the original waveforms.

The sound and vibration signals were divided by a sliding window to form sub-samples, and there was no overlap between each sample. The length of each sample was 1024. All the samples were grouped into seven datasets to test the performance of the proposed approach under different speed conditions. Table 1 shows a detailed description of each dataset. The training and testing set was in the same speed conditions for datasets A1 and A2. Dataset A1 contained four different speed conditions. For each health condition, 100 samples were randomly chosen for each speed. Such a combination of multiple speed conditions is commonly used in many examples in the literature to form datasets for method testing under speed-varying conditions or nonstationary conditions [17,20]. Decreasing the speed instead of constant speeds is considered in dataset A2. For each health condition, 400 samples were randomly chosen, which covered a speed range from 40 Hz to 10 Hz. In addition, more generalized speed-varying conditions were taken into consideration in this paper to test the performance of the proposed F-MSCNN and to verify its generalization ability and stability. Datasets B1-B3 consisted of the same training samples as dataset A1, which were under limited rotation speeds of 10 Hz, 20 Hz, 30 Hz, and 40 Hz. The corresponding testing samples were under other speeds unknown in the training process (dataset B1) or under time-varying speeds at different decreasing rates (dataset B2 and B3). The training and testing samples in dataset B4 were all collected at decreasing speeds, but the decreasing rate varied. The testing set in Table 1 included new datasets that had not been seen before during the training and model selection steps. When training and tuning the hyperparameters of the model, the datasets in the training set in Table 1 were split into two pieces, including a training dataset and a validation dataset. A total of 75% of the datasets were randomly sampled without replacement and put together to train the model. The remaining 25% was used to evaluate the model performance to obtain the best hyperparameters. Then, the well-trained model was finally evaluated by the unseen testing set to obtain the diagnosis accuracies.

### 4.2. Diagnosis Results under Different Speed-Varying Conditions

The vibration and sound signals in each sample were first combined together as an input matrix with a size of 1024 × 2 according to the proposed method. An F-MSCNN model was built and trained using the input matrices obtained from the training samples. The architecture parameters of the proposed F-MSCNN are displayed in Table 2. The output size in Table 2 indicates the output width × output depth. The algorithm was implemented by Python using a computer with Intel Core i7-8700 CPU @ 3.2 GHz and 16 GB RAM. 

**Figure 6:**
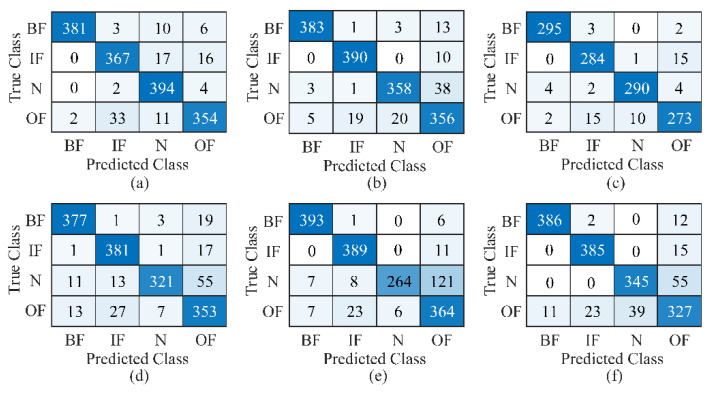
Confusion matrix of F-MSCNN on dataset (**a**) A1, (**b**) A2, (**c**) B1, (**d**) B2, (**e**) B3, and (**f**) B4.

The diagnosis accuracies on six datasets are listed in Table 3. The learning rate was set to 0.0005 during the training process. The hyperparameters of each dataset were carefully optimized to guarantee the best performance of the proposed model. The diagnosis results in Table 3 are average values of 10 times the running. It shows that the proposed F-MSCNN model was effective in the fault diagnosis of rolling bearings under various types of speed-varying conditions with high accuracy and low deviation. The proposed model uses a simple structure that directly works on the raw sound and vibration signals and shows good adaptation ability when crossing different speed domains. The classification accuracies were above 93% for datasets A1 and A2 when the testing samples were in the same working conditions as the training samples. The information on working speeds was well learned during the training process. The mean accuracy was still high at 93.38% in dataset B1 when the testing samples were in the new working speeds, which proves the speed generalization ability of the proposed model. The accuracy results slightly decreased to about 88% when the time-varying speeds instead of constant speeds were considered in the testing samples. It is reasonable that the nonstationary factors were stronger. Figure 6 displays the confusion matrix of F-MSCNN on different datasets. For simplicity, one time from the testing results was taken as an example, and the classification rate of each label could be clearly seen and compared in Figure 6.

#### 4.2.1. Discussion on F-MSCNN Parameters

The parameters of F-MSCNN were decided by trial and error to obtain a relatively better diagnosis performance for all the datasets. When adjusting the parameters, some training and validation samples were used to optimize the parameters. Once the parameters were optimized and selected, they were fixed to evaluate the model performance by the testing samples. 

**Figure 7:**
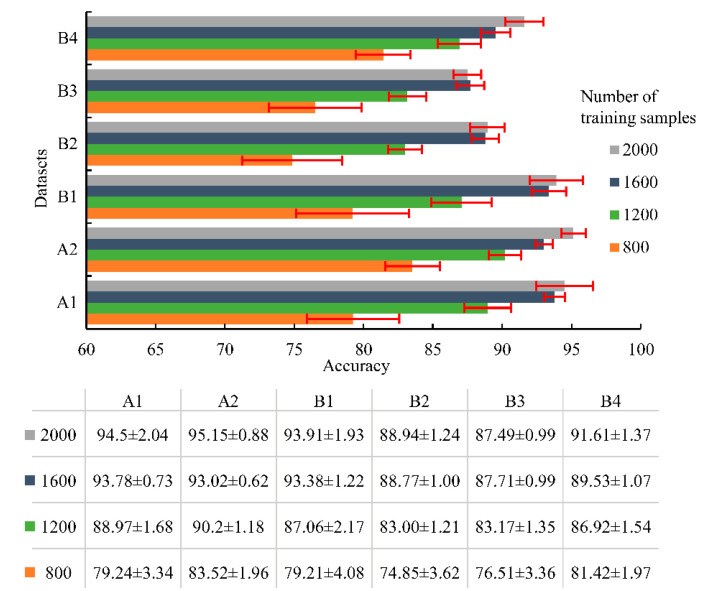
The testing results (mean accuracy ± standard deviation) of different datasets with respect to different numbers of the training samples.

The influence of the training samples on the diagnosis results was carried out in this paper. The mean testing accuracy and standard deviation of different datasets with respect to different numbers of training samples are shown in Figure 7. The mean accuracy ± standard deviation is described by the bar chart with error intervals shown as red lines. Overall, the testing accuracy increased when more samples were available for training, but the increasing rate slowed down sharply when the number of training samples was over 1600. Although the accuracy was higher for 2000 training samples, the standard deviation was higher accordingly, which meant that the performance of the model was not stable. Considering the efficiency and stability, the number of the training samples was set to be 1600 for all the datasets in this paper.

**Figure 8:**
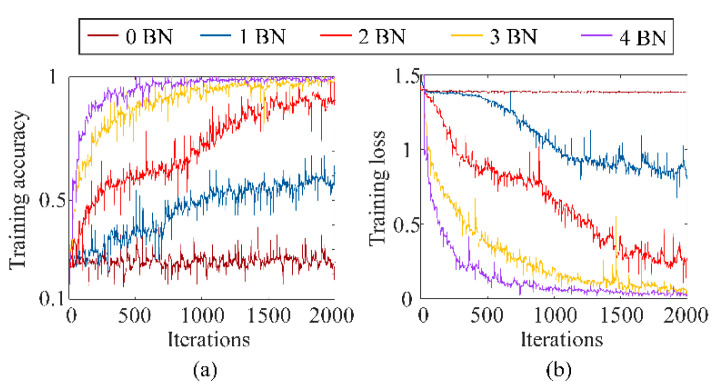
The influence of BN times on the (**a**) training accuracy and (**b**) training loss.

Figure 8 shows the influence of the BN operation on the training accuracy and training loss using dataset B1 as an example. The model tended to converge faster with more times with the BN operation. Additionally, the training accuracy was higher, and the training loss was smaller when adding BN to every convolution layer. 

#### 4.2.2. Visualization Analysis

The proposed F-MSCNN model was able to work directly on the raw signals and learn the features automatically. However, CNN is generally regarded as a black box. The ability to explain the inner feature learning mechanism of the network is worth noticing with further study.

**Figure 9:**
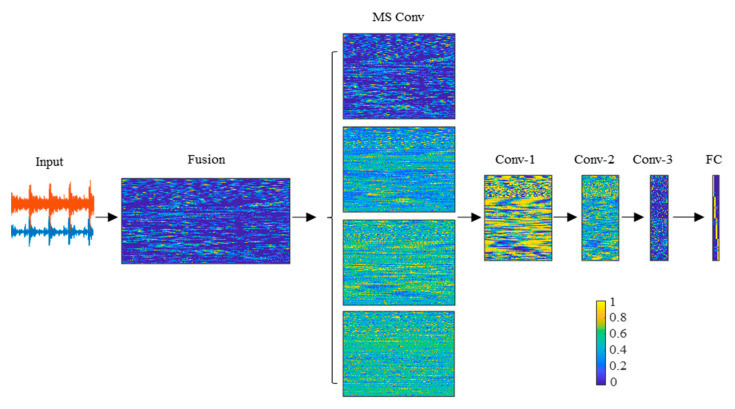
Visualization of the activations of the convolutional layers. Yellow means maximum activation while dark blue represents the unactivated neuron.

The activations of the convolutional layers are visualized in Figure 9 to see the reactions of neurons in different layers. The input is the 1200 testing samples in dataset B1. The activation matrix corresponding to one channel is displayed by each layer for simplicity. It can be seen that the activation map of the fusion layer is vague, and the active neurons were dispersive and irregular. In the MS Conv layer, different neurons were activated by the convolution kernel of different scales, which means that the multi-scale kernel was able to learn more comprehensive features. Additionally, most neurons in the Conv-1 were active so that the information of faults, working conditions in sound, and vibration signals could all be well learned. After several rounds of convolution, representations of four classes in the FC layer could be well-distinguished. Therefore, different health conditions of the rolling bearings could be recognized even when the working speeds are unknown to the trained model and vary.

**Figure 10:**
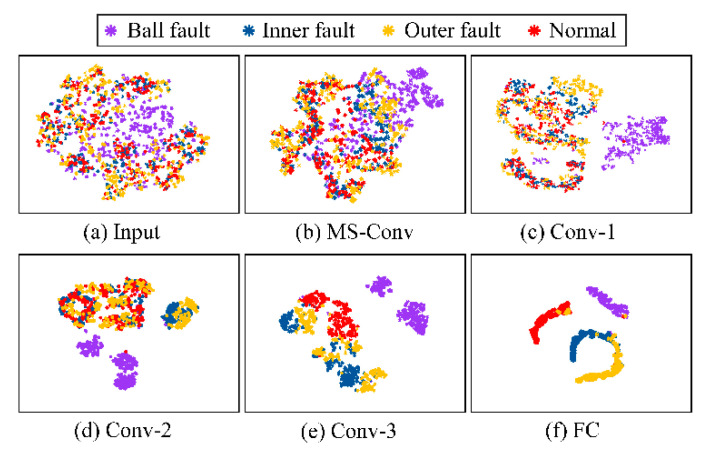
Feature visualization by t-SNE of layer (**a**) Input, (**b**) MS-Conv, (**c**) Conv-1, (**d**) Conv-2, (**e**) Conv-3, and (**f**) FC. The input is the testing samples of dataset B1 and samples of different health conditions are displayed in different colors.

The feature distribution of the test samples was visualized by t-SNE to understand the inner feature learning process qualitatively. Figure 10 displays the learned features of dataset B1. The raw features in Figure 10a are chaotic and complex. Four types of health conditions could not be distinguished from each other. When the layers were deeper, the features of the same class tended to gather more tightly. while the features of different classes were more dispersed from each other, as displayed in Figure 10b–e. The features of the ball fault were easy to divide from the Conv-1 layer, but the other three types still had some overlapped regions. Finally, after four rounds of convolution operation, the boundary between different classes was clear with several clusters in Figure 10f, and the features were well classified. It is noticeable that some outer fault samples were mixed in the cluster of normal conditions, meaning that there were some missing alarms, which is consistent with the diagnosis accuracy of the outer fault (91%). 

### 4.3. Comparison with Other Deep Learning Methods

The proposed F-MSCNN method was compared with other deep learning methods to further demonstrate its effectiveness in signal fusion and multiscale convolutions. The same six datasets are used for comparison, and the results are represented in Table 4. The hyperparameters of each method for each dataset were decided separately by trial and error to obtain a relatively better diagnosis performance. Firstly, the proposed sound and vibration fusion-based model was compared with the multiscale CNN model with only sound (S-MSCNN) or vibration signal (V-MSCNN) as the input. The architecture of S-MSCNN and V-MSCNN were almost the same as F-MSCNN, except for the input, which used single sensors, so there was no fusion layer but a one-dimension convolutional layer instead. The results show that the process of fusion could obtain obviously better accuracy. Additionally, using the vibration signals in this experiment tended to obtain a better performance than the sound signals. Secondly, the proposed F-MSCNN model was compared with the traditional CNN model retaining the fusion process (F-CNN). Instead of a multiscale convolutional layer, a convolutional layer with a single kernel of size 15 was constructed. The performance of F-CNN was not bad but still less excellent than the proposed method, with a mean accuracy about 3% lower than all the datasets. However, the original CNN with a fusion layer showed better diagnosis results than the original CNN with multiscale convolution, which meant that fusion of the sound and vibration signals attributed more to the enhancement of the accuracy in this experiment. Finally, original CNN with only sound (S-CNN) or vibration (V-CNN) signals was also studied in this paper. Without fusion or a multiscale process, S-CNN and V-CNN could not distinguish different faults under varying speeds. The proposed F-MSCNN was effective in fault diagnosis of rolling bearings with high accuracies and strong stability and had the capability of generalization.

The average computational time and the number of trainable parameters for different methods are listed in Table 5, using dataset A2 as an example. The training time is the average time it took different models to train and obtain the best hyperparameters. The testing time is the average time it took these models to predict the label of one testing sample. Fault diagnosis using machine learning methods usually consists of pre-training offline and testing online. A large amount of historical data with labels was used for training to obtain a model which could determine the accuracy of the diagnosis. The proposed method took about 490 s to train, which is time-consuming compared with other methods. Additionally, the number of trainable parameters for F-MSCNN was the largest. The training process required an iterative calculation of gradients and the updating of weights, so the proposed method required more training time with a more complex structure. However, the pre-training process could be completed offline in advance. More attention needs to be paid to the average testing time required for the online diagnosis. These methods all give relatively fast results. Though F-MSCNN shows a 0.06 s lag compared with the fastest one-signal CNN, it is acceptable in industrial applications. The average testing time could be shorter with a better computer.

The proposed F-MSCNN was also compared with the long short-term memory method (LSTM) [40] and the k-nearest neighbors (KNN) [41] with raw signals as the input. The results are shown in Table 6. The hyperparameters of KNN were automatically optimized using the Matlab function. The structure and hyperparameters of LSTM were optimized by trial and error to obtain relatively better diagnosis performance. The results of KNN, which is a traditional machine learning method, are not so convincing. On the one hand, sound and vibration signals were not fused as with F-MSCNN but were simply spliced as the input. On the other hand, the datasets that were used contained various working speeds and four fault types. Using dataset A1 as an example, through 1600 training samples, there are only 100 samples for one speed per fault type. The traditional method may have trouble dealing with such small samples. In this paper, a multiscale convolutional operation is used to gather a different scale of features. The LSTM, as a deep learning method, showed much better results compared with KNN. As the network went deeper, more information could be extracted automatically. However, the diagnosis accuracy dropped below 90% when the testing samples were at different speeds from the training samples. The proposed MSCNN tended to have a strong adaptation performance under speed-varying conditions.

## 5. Conclusions

This paper proposed an improved model named F-MSCNN for the fault diagnosis of rolling bearings under speed-varying conditions using sound and vibration signals. The aim was to classify different types of faults accurately when the speeds of the testing samples were nonstationary and unknown to the trained model. F-MSCNN added a fusion layer and a multiscale convolutional layer to the original CNN to enhance the generalization ability by combining health conditions and working conditions information from different sensors and integrating multiscale features. F-MSCNN provided a simple end-to-end fault diagnosis framework that worked directly on raw sound and vibration signals without hand-crafted feature extraction. The sound and vibration signals were firstly fused by a two-dimension convolution to extract the common and distinctive features. The fused signals are then convolved by multiscale kernels to represent the features of various speeds.

The proposed method was tested by the sound and vibration data from a rolling bearing test bed. Six datasets are constructed to test the effectiveness of the model under different speed-varying conditions. Not only multiple speeds were considered, but speeds fluctuating with time were also studied. The speeds of the testing samples could be the same as the training samples or more strictly restricted to new working conditions unknown to the trained model. The results show that the proposed method had a strong adaptation ability for different speed domains and could achieve a high and stable diagnosis accuracy under different working speeds. The proposed method was also compared with other CNN-based methods and other machine learning methods with higher accuracy, better stability, and strong robustness to the change in speeds. In addition, to display the inner feature leaning mechanism of the network, visualization analysis was performed, including visualizing the activations of all the convolutional layers and feature distribution.

Further work will consider other changeable working conditions, such as the working loads, and conduct experiments on other objects, including the gearbox, to expand the scope of application of the model. The performance of the proposed model could be further explored under compound faults using a blind dataset. An example of a blind test is shown in Reference [42]. Additionally, more efforts could be made to improve the performance of the proposed model further, especially when the speeds are changed with time.

## Figures and Tables

**Table 1:** Description of the datasets.

Datasets	Health Conditions	Training Set		Testing Set	
		Speed (Hz)	Number of Samples	Speed (Hz)	Number of Samples
A1	N/IF/BF/OF	10/20/30/40	1600	10/20/30/40	1600
A2	N/IF/BF/OF	40 to 10 (1 Hz/s)	1600	40 to 10 (1 Hz/s)	1600
B1	N/IF/BF/OF	10/20/30/40	1600	15/25/35	1200
B2	N/IF/BF/OF	10/20/30/40	1600	40 to 10 (1 Hz/s)	1600
B3	N/IF/BF/OF	10/20/30/40	1600	40 to 10 (0.5 Hz/s)	1600
B4	N/IF/BF/OF	40 to 10 (1 Hz/s)	1600	40 to 10 (0.5 Hz/s)	1600

**Table 2:** Architecture parameters of the proposed F-MSCNN.

Layer	Kernel Size	Kernel Count	Stride	Output Size	Activation Function
Input	-	-	-	1024 × 2	-
Fusion	16 × 2	16	1	1009 × 16	ReLU
MS Conv	[5, 15, 25, 1]	16	2	505 × 64	Sigmoid
Pooling	2	-	2	252 × 64	-
Conv_1	10	16	1	243 × 16	Sigmoid
Pooling_1	2	-	2	121 × 16	-
Conv_2	10	32	1	112 × 32	Sigmoid
Pooling_2	2	-	2	56 × 32	-
Conv_3	10	64	1	47 × 64	ReLU
Pooling_3	2	-	2	23 × 64	-
FC (Output)	4	-	-	4 × 1	Softmax

**Table 3:** Diagnosis results for different datasets.

Datasets	A1	A2	B1	B2	B3	B4
Mean accuracy (%)	93.78	93.02	93.38	88.77	87.71	89.53
Standard deviation (%)	0.73	0.62	1.22	1.00	0.99	1.07

**Table 4:** Diagnosis results of different CNN-based methods.

Method	Mean Accuracy ± Standard Deviation (%)					
	A1	A2	B1	B2	B3	B4
F-MSCNN	93.78 ± 0.73	93.02 ± 0.62	93.38 ± 1.22	88.77 ± 1.00	87.71 ± 0.99	89.53 ± 1.07
V-MSCNN	87.50 ± 2.99	90.20 ± 1.38	87.34 ± 2.53	82.04 ± 1.70	78.32 ± 1.34	86.25 ± 1.77
S-MSCNN	81.61 ± 2.20	89.67 ± 3.25	64.98 ± 2.04	74.58 ± 2.28	73.31 ± 1.97	83.28 ± 3.68
F-CNN	91.94 ± 0.88	90.97 ± 1.89	90.16 ± 2.16	85.29 ± 1.72	84.94 ± 1.29	86.26 ± 3.12
V-CNN	83.19 ± 1.77	86.48 ± 0.99	83.93 ± 1.56	76.64 ± 2.15	76.67 ± 1.76	81.57 ± 1.38
S-CNN	73.46 ± 3.93	86.69 ± 2.30	67.58 ± 3.51	71.42 ± 3.27	70.15 ± 3.29	79.17 ± 2.70

**Table 5:** Diagnosis results of different deep learning methods.

Method	F-MSCNN	V-MSCNN	S-MSCNN	F-CNN	V-CNN	S-CNN
Training time (s)	491.67	193.25	193.04	190.10	83.18	83.23
Testing time (s)	0.18	0.15	0.14	0.13	0.12	0.12
Number of Trainable parameters	54,564	54,308	54,308	38,804	42,884	42,884

**Table 6:** Comparison with other machine learning methods.

Method	Mean Accuracy (%)					
	A1	A2	B1	B2	B3	B4
F-MSCNN	93.78	93.02	93.38	88.77	87.71	89.53
KNN	59.97	48.88	52.33	57.73	59.41	48.56
LSTM	91.37	90.62	85.33	79.69	80.75	89.12

## Data Availability

Not applicable.
